# Increasing trends in childhood overweight have mostly reversed: 30 years of continuous surveillance of Slovenian youth

**DOI:** 10.1038/s41598-020-68102-2

**Published:** 2020-07-03

**Authors:** Maroje Sorić, Gregor Jurak, Saša Đurić, Marjeta Kovač, Janko Strel, Gregor Starc

**Affiliations:** 1 Faculty of Sport, University of Ljubljana, Gortanova 22, Ljubljana, Slovenia; 2 Faculty of Kinesiology, University of Zagreb, Zagreb, Croatia

**Keywords:** Health policy, Epidemiology

## Abstract

The aim of this study is to describe trends in overweight and obesity among Slovenian youth for the period 1989–2018. Nearly all schoolchildren in Slovenia had their height and weight measured annually, which lead to a total of 6,738,510 data-points during the 30-year period. The IOTF cut-off points and Joinpoint regression were used to examine annual percent change (APC) in overweight and obesity prevalence across 3 age groups (7–10, 11–14 and 15–18 years). Obesity approximately tripled, while overweight doubled between 1989 and late 2000s in both genders. Since then overweight has been steadily decreasing in all 3 age groups for boys and in 7–10-year-old girls. Obesity has also been declining since 2009, but only in the youngest boys and girls (APC = − 1.9, 95% CI = − 3.2 to − 0.6 and APC = − 1.6, 95% CI = − 3.0 to − 0.2, respectively). Unfavourable trends were noted only in 15–18-year-old girls, with obesity rising at an unchanged rate over the past 30 years (APC = 4.8, 95% CI = 4.5–5.1). Overweight and obesity among Slovenian youth has increased dramatically over the last 3 decades. Still, during the last decade this rise has been reversed or at least stopped. This reversal of trends was more marked in boys than in girls, and in young children compared to adolescents.

## Introduction

Obesity in children has been linked to both short^1^ and long term adverse health outcomes^2^. Moreover, childhood obesity tracks well into adulthood^3,4^, and leads to risks of premature mortality^5^. Childhood obesity has increased dramatically during the last few decades of the twentieth century, especially in developed countries^6^. At that time, childhood obesity was recognised as public health priority what led to diverse preventive strategies being implemented worldwide. The WHO European Charter on counteracting Obesity was adapted in 2006 with the goal to achieve visible progress in the next 4–5 years and to reverse the trend by 2015 at the latest^7^. Yet, obesity continued to rise in low- and middle-income countries in the last 2 decades^6^. At the same time, both overweight and obesity among children have levelled-off in high-income English-speaking countries and most of the Western European countries^6,8^. Still, to date no country has provided reliable evidence on the achievement of the reversal goal of the 2006 Charter^9^.

Unlike in most developed countries, among Slovenian children a steady increase in overweight and obesity continued in the first decade of the twenty-first century^10^, propelled by lifestyle changes in the period of transition from socialist to capitalist socio-economic system^11^ after the brake-up of Yugoslavia in 1990. Namely, in the first years after the independence we were witnessing a fast advance of obesogenic environment. This was first reflected in the changes of nutrition, and was later accompanied by growing sedentariness and declining physical activity. Annual consumption of chocolate, cocoa, cookies and biscuits rose by almost 60%, from 5.5 kg per person in 1990 to 9.2 kg by 2000^12^, and stabilised afterwards. This went along with changes in sedentariness due to proliferation of screen technologies in the households. The share of households with colour TV grew from 72% in 1990 to 96.3% in 2000 , while the share of households with personal computers increased from 12% in 1991 to 46% in 2000, and to 70% in 2010^13,14^. In the same period, the household ownership of bicycles fell from 68.5% in 1990 to 60.4% in 2000^12^. This growing problem has not been adequately addressed until late 2000s, when several nation-wide initiatives targeting both physical activity and nutrition were introduced within the educational system. These efforts were focused on school setting and included directives on school diets that prohibited foods with little nutritional value and accentuated healthy dietary choices, delivery of 2 extra hours of physical education per week and provision of more opportunities for physical activity within the school extra curriculum programme. The initiatives received a lot of media attention, making the fight against childhood obesity an important public concern and increasing awareness among both the children and their parents. However, the trends in childhood obesity in Slovenia in the following period have not been reported. Here, we use an unprecedented amount of data for a single country to describe 30-year trends in childhood overweight and obesity among 7–18-year old youth in Slovenia.

## Methods

This study is based on a large data set collected through the SLOfit—the Slovenian national surveillance system of somatic and motor development of school children between 6 and 18 years of age^15^, which has been continuously monitoring overweight and obesity over the last 3 decades. The SLOfit test battery incorporates anthropometric measurements and fitness tests^15^. The monitoring was implemented in 1982 and after 6-year testing period became compulsory for all Slovenian schools in the school year 1987/1988. This study is based on the data from 1989 to the most recent survey performed in 2018. The number of participants over this interval averaged 224 617 individuals per year, leading to a total of 6,738,510 data points during the 30-year period. During this time the SLOfit monitoring system has been including around 95% off all primary-school children and around 60% of secondary-school youth. The exact numbers of participants across the study period are shown in Table 1.

**Table 1:** Number of participants in the national SLOfit monitoring system across study years.

Year	7–10-year-olds		11–14-year-olds		15–18-year-olds		Total
	Boys	Girls	Boys	Girls	Boys	Girls	
1989	38,067	35,940	40,213	38,649	28,287	32,068	213,224
1990	44,124	41,771	50,874	48,505	34,387	38,347	258,008
1991	43,946	42,309	52,063	49,168	34,791	38,393	260,670
1992	43,409	42,084	52,349	49,683	39,616	43,180	270,321
1993	45,865	44,114	55,019	52,609	42,230	45,185	285,022
1994	45,710	44,306	54,710	52,167	44,894	46,651	288,438
1995	44,615	43,452	52,973	50,957	44,995	46,492	283,484
1996	44,961	43,390	52,027	49,983	44,126	45,088	279,575
1997	42,851	40,588	48,121	45,545	36,930	34,490	248,525
1998	42,007	39,858	47,092	45,219	37,735	36,822	248,733
1999	40,283	37,828	46,646	44,770	36,255	37,258	243,040
2000	38,666	36,368	45,978	43,938	37,521	36,000	238,471
2001	37,137	35,330	44,873	42,945	36,618	35,773	232,676
2002	36,051	34,315	43,698	41,479	32,916	32,959	221,418
2003	36,174	34,223	42,241	39,996	36,392	36,006	225,032
2004	36,253	34,476	40,386	37,943	36,053	35,094	220,205
2005	35,776	33,999	39,128	37,102	35,022	33,402	214,429
2006	34,854	32,614	37,272	35,305	32,809	30,294	203,148
2007	34,102	32,096	36,317	34,180	27,555	29,089	193,339
2008	34,246	32,195	36,237	34,150	28,450	26,924	192,202
2009	34,484	32,239	35,806	33,846	28,405	27,876	192,656
2010	34,211	32,133	35,248	32,954	26,798	26,532	187,876
2011	34,015	32,256	34,726	32,569	27,170	26,321	187,057
2012	34,091	32,263	34,669	32,546	27,234	25,994	186,797
2013	34,683	32,572	34,141	32,192	24,849	25,461	183,898
2014	35,505	33,821	34,309	32,402	26,722	25,530	188,289
2015	36,860	35,010	34,307	32,463	26,178	25,377	190,195
2016	39,041	37,104	34,501	32,426	27,505	26,228	196,805
2017	40,671	39,016	34,761	33,037	27,231	26,097	200,813
2018	42,033	39,966	35,661	33,813	26,697	25,994	204,164
Total	1,164,691	1,107,636	1,266,346	1,202,541	996,371	1,000,925	6,738,510

Every April the measurements are conducted by physical education teachers in all Slovenian schools according to the uniform official protocol^15^. During the course of their graduate education, physical education teachers are thoroughly educated in anthropometry with level of detail that exceeds the demands of the SLOfit system. All the schools in Slovenia are equipped with the required measurement instruments, including medical scales with stadiometers. Height and weight are measured to the nearest 0.1 cm and 0.1 kg, respectively, and BMI is calculated according to standard procedures. Until 2018 weight that exceeded 100 kg was censored to 99.9 kg, due to administrative limitations (i.e. official record forms demanded the input of weight in dkg by 3 digits). In 2018, weight in dkg was recorded with 4 digits, allowing to record the actual weight above 99.9 kg. During the measurements, children are barefoot and wear light sports clothing. After the school-based measurements, the data are sent to the Faculty of Sport at the University of Ljubljana where they are checked, cleaned, analysed and the feedback reports are sent back to schools for every individual child. The prevalence of overweight and obesity in this study was calculated based on the extended, age and gender-specific, IOTF criteria^16^. In the overweight category, we included only children with overweight, but not children with obesity or severe obesity). Similarly, obesity includes only children with obesity and not children with severe obesity.

### Statistical analyses

The standardized rate trends by sex and age groups were analysed using Joinpoint Regression Program v. 4.7.0.0 (Information Management Services, Inc., Calverton, MD)^17^. We pooled several ages in order to form three age groups: children (7–10 years), early adolescents (11–14 years) and middle adolescents (15–18 years). Joinpoint regression model and grid-search method were applied in order to calculate average annual percent change (APC) in the prevalence of overweight and obesity in the period 1989–2018. Furthermore, we used joinpoint regression analysis to identify year when changes occurred in the linear slope of the time-based trend. When the rate changed significantly, best fitting points (so-called joinpoints) were chosen. Thus, through applying joinpoint regression analysis, we identified the moment when the change has occurred in the trend, as well as the magnitude of the increase or decrease noticed in interval by estimating average APC. All analyses were stratified by gender and Alpha was set at *p* = 0.05.

### Ethical considerations

According to the national legislation, in primary schools parents are required to sign an informed consent form for their child’s measurements to be analysed and included in the SLOfit database, while in secondary schools it is the youngsters who sign informed consent. Parents and children are informed about the study protocols and their right to withdraw their consent at any time. All procedures in this study were in accordance with the Declaration of Helsinki, and were approved by the National Medical Ethics Committee of the Republic of Slovenia (ID 102/03/15).

## Results

All in all, in the last 30 years overweight increased from 11.8 to 18.9% among Slovenian boys and from 11.4 to 17% in girls, while obesity rose from 1.9 to 5.9% in boys and from 1.7 to 4.7% in girls. In 1989 severe obesity was present in under 0.3% of boys or girls, while in 2018 it was recorded in 1.3% of the boys and 1.1% of the girls. The 30-year increase was larger in boys compared to girls for all three categories of excess weight (7.1 percentage points vs. 5.6 pp for OW, 4 pp vs. 3 pp for OB and 1.0 pp vs. 0.8 pp for severe OB).

**Figure 1:**
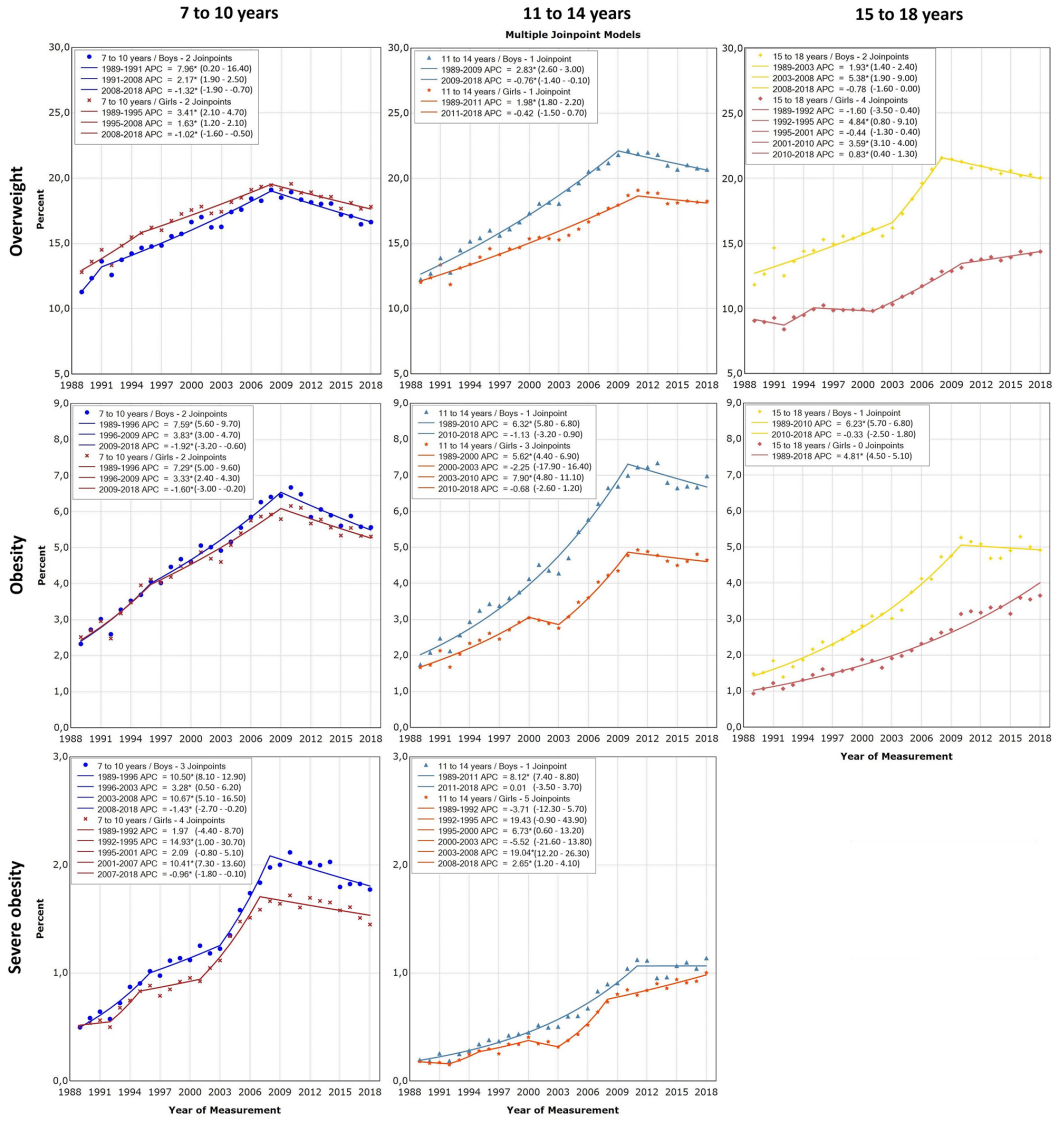
Time trends in the prevalence of overweight, obesity and severe obesity between 1989 and 2018 depicted by sex, across three age groups: 7–10 years, 11–14 years and 15–18 years. Numbers in the legends denote annual percent increase -APC (95% confidence interval) over a specific period.

More detailed depiction of the temporal trends in the prevalence of overweight, obesity and severe obesity between 1989 and 2018 are shown in Fig. 1, together with the results of the joinpoint analyses for difference in trends over time.

Inspection of Fig. 1 reveals trends that are rather consistent across gender and age groups in the first 2 decades, but diverge afterwards. Overall, both overweight and obesity increased more in boys than girls, resulting in increasing gap between genders, especially in the two older age groups. In 1989, the prevalence of overweight was rather similar across age groups and ranged from 9.1% in 15–18-year old girls to 12.8% among 7–10-year old boys. At the same time, the prevalence of obesity and severe obesity also did not differ much between groups and was lower than 2.5% (obesity) and 0.5% (severe obesity) in every age group-gender combination. After that, the prevalence of all 3 categories of excess weight increased in all age groups and in both genders until late 2000s, although seemingly at a somewhat different rate. Generally, the rise was larger in boys than in girls, with differences in APC ranging from about 0.5% per year in 7–10-year-olds to more than 1.5% per year for obesity in 15–18-year-olds. During that period the prevalence of overweight increased steadily in all groups except for girls aged 15–18 years, where it rose by 4.8% per annum (95% CI = 0.8–9.1) from 1992–1995 and by 3.6% per annum (95% CI = 3.1–4.0) in 2001–2010, but was stable meanwhile. On the other hand, boys in this age group exhibited the highest rise in the prevalence of overweight, reaching 5.4% per year (95% CI = 1.9–9.0) from 2003–2008. At the same time, the increase in the prevalence of overweight was less pronounced in two younger groups of children with the smallest increase over a longer period being observed for 7–10-year-old girls between 1995 and 2008 (APC = 1.6, 95% CI = 1.2–2.1). The increase in the prevalence of obesity until late 2000s was even larger than for overweight, peaking at 7.9% (95% CI = 4.8–11.1) rise each year from 2003–2010 in 11–14-year old girls, followed by 7.6% (95% CI = 5.6–9.7) and 7.3% (95% CI = 5.0–9.6) yearly increase between 1989 and 1996 in 7–10-year old boys and girls, respectively. Finally, the prevalence of severe obesity showed the largest number of joinpoints over the period between 1989 and late 2000s. Periods of stagnation were interlinked with periods of large increase. The exception were 7–10-years-old boys in whom the rise was noted over the whole 20-year period (1989–2008) at rates surpassing 10% per annum for 1989–1996 and 2003–2008. This led to more than 2% of 7–10-years-old boys being recorded with severe obesity in 2008.

Since late 2000s, overweight appears to have levelled-off in adolescent girls and declined in male adolescents and younger boys and girls. More specifically, in 7–10-year old boys and girls overweight decreased by 1.3% per annum (95% CI = − 1.9 to − 0.7) and 1% per annum (95% CI = − 1.6 to − 0.5) between 2008 and 2018, respectively. Similarly, in 11–14 and 15–18-year old boys we observed an annual decrease of 0.8% in overweight between 2009–2018 and 2008–2018, respectively (95% CI = − 1.4 to − 0.1 and − 1.6 to 0.0, for 11–14 and 15–18-year old boys, respectively). On the other hand, in girls the rise in overweight has stopped in the 11–14-year old group (APC = − 0.4, 95% CI = − 1.5 to 0.7), but has continued, albeit at a slower rate, in 15–18-year olds (APC = 0.8, 95% CI = 0.4–1.3).

Like for overweight, during the last 9–10 years the increase in obesity has been reversed or stopped in all age group-gender combinations, except for 15–18-year old girls. The rates have been decreasing from 2009 to 2018 in the youngest boys and girls (APC = − 1.9, 95% CI = − 3.2 to − 0.6 and APC = − 1.6, 95% CI = − 3.0 to − 0.2, respectively). In addition, a trend toward a reversal in obesity prevalence was observed among 11–14-year boys and girls and 15–18-year old boys in 2010–2018 period (Fig. 1). Conversely, in 15–18-year old girls the increase in obesity has been continuous during the past 30 years (APC = 4.8, 95% CI = 4.5–5.1).

Similar to what has been described for OW and OB, the largest changes in trends in severe obesity were also observed among the youngest children. After the notable rise in the first 20 years of the observation period, the trends have started to change around 2007/2008. Since then, severe obesity has been decreasing in both 7–10-year-old boys and girls (APC = − 1.4, 95% CI = − 2.7 to − 0.2 and APC = − 1.0, 95% CI = − 1.8 to − 0.1, respectively). At the same time in in 11–14-year-old girls the increase in severe obesity has slowed down (APC = 2.7, 95% CI = 1.2–4.1), while a bit later, around 2011, it has levelled-off in 11–14-year-old boys (APC = 0.0, 95% CI = − 3.5 to 3.7).

## Discussion

Using an unprecedented amount of data for a single country, this study assessed 30-year trends in overweight, obesity and severe obesity across a wide age range of Slovenian youth. The main finding of the current study is that after a marked increase in the prevalence of overweight and obesity between 1989 and late 2000s, in the last 8–11 years a clear reversal of trends was observed in most age and sex groups. Specifically, over that period overweight steadily decreased in boys of all ages and in 7–10-year old girls, while it continued to increase only in 15–18-year old girls, albeit at a slower rate compared to the first 20 years of observation. Next, a permanent decrease in obesity was also noted over a comparable period, although this was unambiguous only in the youngest boys and girls, and a bit less certain in early adolescent boys. The oldest girls were the only group where no change in trends of obesity prevalence was observed, with unceasing increase being observed over the whole 30 years of observation.

Our findings diverge from contemporary global trends. A recent large analysis of global trends reported on a continued increase in the average BMI trend globally, with the stabilisation of increasing trends during the last few decades in high-income countries^6^. On the other hand, the findings of single-country population studies are not unequivocal. Specifically, the prevalence of childhood overweight and obesity generally stabilized in Spain^18^, Greece^19^, and Korea^20^, while at least some decrease was observed in Germany^21^, Portugal^22^, Japan^23^ and Italy^24^. Nonetheless, it should be noted that the observed declines were generally not consistent across age groups and time. For example, trend analysis in Keß et al.^21^ study demonstrated that the prevalence of overweight and obesity in 326,834 German children and adolescents (4–16 years) significantly decreased from 2005 to 2010 in 4–12 year-olds, while at the same time an increase was observed in adolescent boys (12–16 years). In addition, these trends did not continue in the following period, from 2010 to 2015. Similar trends were noted in Portugal between 2002 and 2010, when the prevalence of overweight and obesity decreased in 11–13-year-olds, while simultaneously increasing among 15–17-year-olds. Thus, no change in overweight and obesity prevalence was evident overall^22^. Unlike all the previous studies, the results of a recent study from Italy are in line with the present study. Based on a nationally representative sample (about 45,000 children), a decline in the prevalence of overweight and obesity was reported in the period from 2008 to 2016^24^. The magnitude of this decline was larger than noted in this study, although comparison is hindered by the use of different cut-off points for the definition of obesity.

Notwithstanding the optimistic finding of the present study that overweight and obesity have been decreasing in Slovenian children in the last decade, it has to be emphasised that the levels are still disappointingly high. In 2018 the overall prevalence of overweight (including obesity) among Slovenian youth amounted to almost 26% in boys and 23% in girls what is slightly higher than the average reported in the most recent IOTF cut-off based global estimates for developed countries^25^. Note that 30 years ago, the abovementioned prevalence corresponded to around 14% for both boys and girls, while in 2006 these values were similar to the present (26% and 21% boys and girls, respectively).

A marked effect of age on trends in overweight and obesity was evident in the current study. Children (7–10 years) and early adolescents (11–14 years) both experienced a steeper increase in overweight and obesity until late 2000s compared to middle adolescents (15–18 years), but also a more marked reversal in trend afterwards. Overall, this study showed that the burden of excess weight in Slovenia during the last 30 years was more pronounced in preadolescent and early adolescent children compared to their peers in middle adolescence. Obesity was more prevalent in the younger age groups compared with the older ones throughout the last 30 years in both genders. The difference in the prevalence of obesity between the two youngest age groups and the oldest age group widened until late 2000s and decreased since. On the other hand, age differences in trends of overweight were more complex. In 1989 overweight rates among boys were comparable across all age groups, but since then overweight increased more in 11–14-year old early adolescents than in the other two age groups. Notwithstanding the decreasing trend in this age group during the last 5 years, at present the overweight rates are still the highest in the 11–14 olds. In girls, however, younger age groups exhibited higher overweight rates than adolescents since the beginning of the observed period. This gap has been widening in the first 2 decades, but narrowed afterwards, due to a decrease in the youngest age group and a continued increase in 15–18-year olds. If these trends continue, obesity will be more prevalent in adolescent girls than in their younger peers by 2024. All in all, our findings are in line with the observations from the U.K^26^, France^27^, the U.S.^28^, Mexico^29^ and Australia^30,31^ where trends were also less favourable in adolescents compared to younger children. Taken together, these findings indicate that current strategies are ineffective for adolescents and that new strategies tailored specifically for this age are urgently needed.

In the current study the increase in obesity seen from 1989 until late 2000s was more pronounced in boys compared to girls across all age groups. This is line with the findings of the largest global study that, from 1975 to 2016, global prevalence of obesity increased more in boys than in girls^6^. On the other hand, the decrease in both overweight and obesity seen in the last 10–11 years in 7–10-year old children was also more pronounced in boys than in girls. In addition to this, the trends for overweight and obesity in the other two age groups were also more favourable for boys than girls. Similar patterns across genders were shown in Germany^21^ and Italy^24^. In contrast to these findings, several previous national studies report that the plateauing of obesity was more marked for girls than boys^32^. Likewise, data from New Zealand, England, Northern France and Sweden indicate larger changes in obesity among young girls as compared to boys, but no gender difference among adolescents^8,27^. All in all, it seems that gender differences in susceptibility to obesogenic environments as well as in the response to interventions are driven by numerous socio-cultural factors and vary by region. Further studies are needed to identify such factors more precisely.

Even though the reasons for the reversal in obesity trends in developed countries noted in this and some prior studies are not understood, it has been suggested that major policy changes including interventions aimed at behavioural change may have contributed to this phenomenon^30^. In Slovenia, coordinated public health initiatives aimed specifically at combating childhood obesity started only several years ago. The efforts were focused on providing more opportunity for physical activity within the school extra curriculum programme. At the same time, foods with little nutritional value were excluded from school meals and healthy dietary choices were accentuated. Starting with school year 2010/2011 Healthy Lifestyle intervention program that supplemented the existing 2 or 3 h of physical education per week with 2 extra hours, taught by physical education teachers, was introduced in about 30% of Slovenian primary schools^33^. Although the intervention was not compulsory and did not include all the children in individual schools this program managed to involve around 20% of all 7–14-year old children in Slovenia by 2015. The Healthy Lifestyle initiative focuses on health enhancing physical activity, it is free of charge, open to anyone, but aimed especially at children who are not engaged in out-of-school sporting activities are generally less fit. In the same year the School Meals Act was adopted along with the National Dietary Guidelines^34^, prescribing healthy school meals and banning vending machines from all Slovenian schools. Finally, in 2013 an additional elective school subject Sport was introduced in all Slovenian schools in grades 4–6 and provided an extra hour of physical education per week within the school curricula. All these initiatives also received a lot of media attention which probably resulted in increased awareness of the obesity burden among parents and teachers. Albeit the design of this study precludes inferring causality, two hints point to the possible role of these public health campaigns in the decline of overweight and obesity spotted in this study. First, the evident change in the trends in overweight and obesity is located just around the launching of the first interventions in 2010. Second, all large public health initiatives in Slovenia were targeted at primary school children only, and the decrease in overweight and obesity in this study was indeed confined to primary-school age groups, but was not seen in secondary-school adolescents. Thus, it appears that interventions implemented so far have been effective, but it also seems that the effects are waning in the long term. Therefore, similar initiatives aiming specifically at adolescents are needed for a more sustainable effect on obesity rates. On the other hand, it has to be noted that in 7–10 -year-old children the upturn of trends in all 3 adiposity categories was observed a year or two before the introduction of obesity prevention policies. This divergence was possibly driven by the fact that in the school year 2007/2008 the introduction of 9-year primary school was completed, meaning that all children in this age group entered school at age 6. Earlier entrance to schools was accompanied by earlier benefits incurred from structured physical education classes and other non-curricular physical activity programmes which may have led to reduction in overweight prevalence in this age group.

### Strengths and limitations

Strengths of this study include an unprecedented amount of data for a single country analysis, stemming from an extremely large sample that approaches census data, thus eliminating notable sample bias. In addition, continuous annual surveillance of children during the past 30 years enabled us to use jointpoint regression, a sensitive approach for exploring trends in registry-based data, to examine annual changes in childhood obesity over a long period. Finally, the analyses in the present study are based on measured height and weight, leading to a considerably lower misclassification compared to the one found in studies based on self-report^35^.

On the other hand, several limitations are also worth noting. First, although the study is based on near census data, in joipoint analysis the unit of measurement is year and not individual. Therefore, only 9–11 data points were available after the last joinpoint. In some occasions this resulted in estimates with rather large standard errors and, hence, relatively wide confidence intervals. Therefore, longer time span will be required before we can be confident that some of the noted trends are not just a temporary fluctuation. Second, we used BMI to assess obesity and BMI is known to be an imperfect measure of adiposity^36^. As no measure of body composition were available, changes in the prevalence of obesity and overweight noted in the present study could be attributed to either changes in body fat or lean body mass. Third, participation of secondary-school youth in measurements was constantly lower compared to primary school children (around 60% vs. 95%) and this was especially pronounced for youth attending vocational schools. We have previously shown that the highest prevalence of overweight and obesity was observed in vocational schools. To that end, it is likely that the prevalence of overweight and obesity among secondary-school youth could be higher than reported in this study throughout the studied period. At the same time, this should not hamper the conclusion of the study as the participation rates were very stable over the whole period of the study so temporal trends are unlikely to be affected. Fourth, since the exact weight of children and adolescents weighing over 100 kg has only been recorded since 2018, we were not able to present trends in the prevalence of severe obesity among 15- to 18-year-olds. Next, in the 11–14 age group, the increased precision in recording the weight above 100 kg may have led to a slightly increased trend of severe obesity in 2018, although most children in this age group, whose weight exceeds 100 kg, fall into the category of severe obesity, even if the recording of their weight is limited to 99.9 kg. Last, we did not collect data about children’s socioeconomic status. Low socioeconomic status has been linked to higher prevalence of obesity in developed countries^37,38^ and trends of growing socio-economic disparities in overweight prevalence in other high-income countries have been reported^39,40^. Thus, although we noted that overweight and obesity have been generally decreasing in children and early adolescents in the last 8–10 years, we cannot dismiss the possibility that the disparities still persist, and that in less affluent groups obesity continues to be on the rise.

## Conclusion

Overweight and obesity among Slovenian school-going youth has increased markedly over the last 3 decades. Still, since the late 2000s, a reversal in trends was seen, especially in the youngest schoolchildren. In addition, the observed trends in the last decade were more favourable for boys than girls in all age groups. The fact that detected trends are the least favourable for adolescents, especially adolescent girls in whom overweight and obesity are still on the rise, point to the need for large-scale interventions tailored particularly for these groups. Finally, it should be noted that, notwithstanding the observed decrease during the last 10 years, overweight and obesity rates in Slovenian youth are still very high. Thus, existing public health efforts in combating obesity should continue with unabated intensity. Studies examining future trends are imperative to ascertain whether the decrease in childhood obesity reported in this study will be sustained and to direct future interventions.
